# Photophysical image analysis: Unsupervised probabilistic thresholding for images from electron-multiplying charge-coupled devices

**DOI:** 10.1371/journal.pone.0300122

**Published:** 2024-04-05

**Authors:** Jens Krog, Albertas Dvirnas, Oskar E. Ström, Jason P. Beech, Jonas O. Tegenfeldt, Vilhelm Müller, Fredrik Westerlund, Tobias Ambjörnsson

**Affiliations:** 1 Centre for Environmental and Climate Science, Lund University, Lund, Sweden; 2 Department of Physics and NanoLund, Lund University, Lund, Sweden; 3 Department of Life Sciences, Chalmers University of Technology, Gothenburg, Sweden; University of Electronic Science and Technology of China, CHINA

## Abstract

We introduce the concept photophysical image analysis (PIA) and an associated pipeline for *unsupervised probabilistic image thresholding* for images recorded by electron-multiplying charge-coupled device (EMCCD) cameras. We base our approach on a closed-form analytic expression for the characteristic function (Fourier-transform of the probability mass function) for the image counts recorded in an EMCCD camera, which takes into account both stochasticity in the arrival of photons at the imaging camera and subsequent noise induced by the detection system of the camera. The only assumption in our method is that the background photon arrival to the imaging system is described by a stationary Poisson process (we make no assumption about the photon statistics for the signal). We estimate the background photon statistics parameter, λ_*bg*_, from an image which contains both background and signal pixels by use of a novel truncated fit procedure with an automatically determined image count threshold. Prior to this, the camera noise model parameters are estimated using a calibration step. Utilizing the estimates for the camera parameters and λ_*bg*_, we then introduce a probabilistic thresholding method, where, for the first time, the fraction of misclassified pixels can be determined *a priori* for a general image in an unsupervised way. We use synthetic images to validate our *a priori* estimates and to benchmark against the Otsu method, which is a popular unsupervised non-probabilistic image thresholding method (no *a priori* estimates for the error rates are provided). For completeness, we lastly present a simple heuristic general-purpose segmentation method based on the thresholding results, which we apply to segmentation of synthetic images and experimental images of fluorescent beads and lung cell nuclei. Our publicly available software opens up for fully automated, unsupervised, probabilistic photophysical image analysis.

## Introduction

Optical imaging, the acquisition of images using light in the visible or near visible range, and, in particular fluorescence imaging, has wide-spread biological and biophysical application and is therefore at the heart of science and engineering [1].

On the experimental side, fluorescence imaging experiments have undergone major advances recently. First, experimental platforms have been brought to high-throughput [2–4], which increases demands for automated analysis. Secondly, super-resolution fluorescence microscopy, such as STORM and STED have emerged [5–7]. On the analysis side, deep learning techniques are gaining popularity [8].

The electron-multiplying charge-coupled device (EMCCD) [9] is often used in fluorescence microscopy imaging as it has all the advantages of a camera (compared to detectors that require scanning) and can detect photons even from single fluorophores.

When processing fluorescence images from EMCCD-camera-based setups one has to deal with the unavoidable effects of different sources of noise [10]. These noise sources can be separated into photon noise, which is described by Poisson statistics, and the subsequent camera-induced noise. Fortunately, the process governing the camera noise present in the image counts of images generated by EMCCD cameras has been studied before and is rather well understood [11–13]. The noise model includes Poisson-Gamma distribution for electron multiplication [11], Gaussian read-out noise and also the rounding of the final image counts [14]. Camera noise model parameters are typically estimated using a mean-variance test along with a set of calibration experiments [15], or, using maximum likelihood estimation (MLE) [16].

The over-arching purpose of this study is to shift the focus of common image analysis tasks [17] (such as filtering, edge detection, registration, segmentation, thresholding and compression) from a purely mathematics-algorithm perspective to also utilize the physics for the recorded intensity values to provide *a priori* misclassification rates for different image analysis tasks. To this end, we here introduce the term *photophysical image analysis* (PIA), and we demonstrate how to approach the common problem of image thresholding from a PIA perspective. Image thresholding refers to the task of classifying image pixels as background or signal [18, 19]. Such thresholding techniques can be supervised (manual selection of threshold) or unsupervised, such as the Otsu method [20]. The Otsu method, the workhorse of unsupervised thresholding, works by separating the intensity histogram into two clusters by minimizing the sum of within-class-variances for the pixel intensities in the image. In here, we introduce a new PIA-based image thresholding method which, for the first time, allow us to do *unsupervised* thresholding and at the same time provide *a priori misclassification rates* for general images. We demonstrate that our *a priori* estimates of pixel misclassification rates are close to ground-truth values in synthetically generated images. For completeness, we also introduce a simple heuristic general-purpose segmentation method, which we apply to experimental images of fluorescent beads and labeled cell nuclei. Image segmentation is the process of identifying meaningful signal regions (for instance, objects such as macromolecules or biological cells) in the image.

MATLAB implementations of our PIA methods are made available as a MATLAB package “EMCCD-PIA”, see Data availability statement. Our methods fulfill the requirements demanded in the image analysis in conventional and high-throughput microscopy, namely reproducibility, full automation and control over the error rate in pixel classifications. In addition, our method is computationally fast and robust.

## Materials and methods

In this section we introduce our PIA methodology for unsupervised probabilistic image thresholding. The main novelties of our method appear in the subsections “Estimating the background illumination strength, λ_*bg*_” and “Unsupervised probabilistic image thresholding”. In the first of these subsections, we show how one can estimate λ_*bg*_ from an image which contains both background and signal pixels (at arbitrary fractions and signal-to-noise ratio, SNR). In the second of these subsections we show how to, in addition, estimate the number of background and signal pixels in the image and use this information to estimate misclassification rates when performing image thresholding. Subsection “Theory” contain the necessary theoretical background and subsection “Camera parameter estimations” shows how to, once for a given camera, estimate the camera noise parameters. In the final subsection “Image segmentation” we present a simple heuristic but general-purpose image segmentation method which uses the image thresholding result as input.

### Theory

Under stationary conditions, the photons stemming from light emitting molecules, such as those used in fluorescence imaging, are described by Poisson statistics. This statistics is controlled by a single parameter—the number of emitted photons per time unit. Moreover, sums of random numbers drawn from a Poisson distribution will again yield Poisson statistics. For a detector exposed to light from multiple sources, the number of incoming photons is therefore also described by a Poisson distribution.

The first step in the EMCCD camera response is converting photons to electrons. The number of input electrons *n*_*ie*_ approximately follows a Poisson distribution, such that $p\left(n_{ie}|\lambda \right)=P\left(n_{ie};\lambda \right)$, with the Poisson parameter λ = *Q*Λ + *c*, which is determined by the average number of photons Λ during the exposure time, the quantum efficiency *Q*, as well as the extra spurious charge *c* generated in the conversion process.

In the next step, the electronic signals are typically amplified with a certain *gain* through many electron multiplication steps. Each of these steps introduces noise, and the number of output electrons, *n*_*oe*_, after these steps is usually modeled by a Gamma distribution with shape parameter *n*_*ie*_ and scale parameter *g*, such that [11]

$$p(n_{oe}|n_{ie},g)=\left\{\begin{array}{l} \delta_{n_{oe},n_{ie}},\;\text{without}\;\text{gain}\\ n_{oe}^{n_{ie}-1}\frac{e^{-n_{oe}/g}}{\Gamma \left(n_{ie}\right)g^{n_{ie}}},\;\text{with}\;\text{gain}\;\text{and}\;n_{ie}\geq 1\\ \delta_{n_{oe},0},\;n_{ie}=0 \end{array}\right.$$ (1)

where *g* is the electronic gain factor, Γ(⋅) is the gamma function and $\delta_{n_{oe},n_{ie}}$ is the Kronecker delta-function.

The electrons are then converted to a digital image count *n*_*ic*_ by the readout process. This process is usually modeled by a normal distribution, such that

$$\begin{array}{c} p\left(n_{ic}|n_{oe},f,r,\Delta \right)=\frac{1}{\sqrt{2\pi r^{2}}}\text{exp}[-\frac{{\left(n_{ic}-n_{oe}/f-\Delta \right)}^{2}}{2r^{2}}]. \end{array}$$ (2)

where *f* is the analog-to-digital conversion factor, *r* is the readout noise and Δ is an offset which is added to ensure that *n*_*ic*_ > 0.

Finally, the image count is rounded to the nearest integer to get the rounded image count *n*_*icr*_. The error introduced in this step is commonly assumed to be described by a uniform distribution [14]. Note that rounding was not included in the study by Ryan et al. [16].

The set of distributions above determine the probability mass function (PMF) for the rounded image count, *n*_*icr*_, in terms of the Poisson parameters λ and the chip parameters, *chipParams* = {*g*, *f*, *r*, Δ}. We here make use of the characteristic function, CF (the Fourier transform of the PMF) for *n*_*icr*_. As we show in our derivation (see Sec. S1 in S1 Text), this CF takes a simple analytic form:

$$\begin{array}{c} \left\langle e^{ipn_{icr}}\right\rangle =\text{exp}[-\frac{p^{2}r^{2}}{2}+ip\Delta ]\text{exp}[\lambda (\frac{1}{1-ip(g/f)}-1)]\frac{\text{sin}\;p/2}{p/2}. \end{array}$$ (3)

The PMF for *n*_*icr*_ (here referred to as the EMCCD-PMF) is obtained by the inverse Fourier-transform of Eq (3), see Sec. S2 in S1 Text for details.

The characteristic function given in Eq (3) can, through Taylor-expansions in *p*, be used to calculate cumulants and moments to any order. For instance, the mean is

$$\begin{array}{c} \mu ≔E\left[n_{icr}\right]=\lambda \frac{g}{f}+\Delta ,\; \end{array}$$ (4)

and the variance is given by

$$\begin{array}{c} \sigma^{2}≔\text{Var}\left[n_{icr}\right]=2\lambda (\frac{g}{f}{)}^{2}+r^{2}+\frac{1}{12}. \end{array}$$ (5)

Note that this variance includes the readout noise and the rounding effect (the factor 1/12).

For EMCCD devices one can also turn off the gain (electron multiplication steps). For this case (see Sec. S1 in S1 Text), we instead have:

$$\begin{array}{c} \mu_{\text{nogain}}≔E\left[n_{icr}^{\text{nogain}}\right]=\lambda \frac{1}{f}+\Delta , \end{array}$$ (6)

while

$$\begin{array}{c} \sigma_{\text{nogain}}^{2}≔\text{Var}\left[n_{icr}^{\text{nogain}}\right]=\lambda (\frac{1}{f}{)}^{2}+r^{2}+\frac{1}{12}. \end{array}$$ (7)

### Camera parameter estimation

For our unsupervised probabilistic image thresholding method (see next two subsections), we need the four camera specific parameters, i.e. *chipParams* = {*g*, *f*, *r*, Δ}, as input. We here show how to determine these parameters using a set of calibration experiments. To this end, we adapt the image stacking method [11] as described below. Note that the calibration needs to be done only once for a given camera setting.

We first generate experimental calibration data (see Sec. S3 in S1 Text for experimental details). To that end, we record a stack of images of (non-moving) fluorescent beads with the gain set to its lowest setting or turned off, and the illumination intensity ranging from 0% to 100%. We then record another image stack at a specific gain setting. For each pixel (here labeled by *j*) we then have a time series of image counts. From these time series we compute sample estimates for the mean ${\bar{x}}^{\left(j\right)}$ and for the associate variance *S*^2(*j*)^ for each pixel.

In order to extract the camera parameters, we next perform a mean-variance analysis of the experimental data. For the no-gain case, Eqs (6) and (7) yield a mean-variance relation

$$\begin{array}{c} \sigma_{\text{nogain}}^{2}=\frac{1}{f}\mu_{\text{nogain}}-\frac{\Delta }{f}+r^{2}+\frac{1}{12}. \end{array}$$ (8)

For the case with gain, Eqs (4) and (5), give the mean-variance relation

$$\begin{array}{c} \sigma^{2}=2\frac{g}{f}\mu -2\frac{g\Delta }{f}+r^{2}+\frac{1}{12}. \end{array}$$ (9)

Note that both expressions above yield a linear relation between the variance and the mean. To extract the parameters we then fit straight lines to a variance versus mean plot for the experimental data, i.e. to the dataset consisting of $\left({\bar{x}}^{\left(j\right)},S^{2(j)}\right)$. In this step, we removed 40% intensity value for the gain = 100 setting, as well as bottom and top 1% of pixels from each experiment to reduce the number of outliers. The slope of the linear fit, $y=k_{\text{nogain}}{\bar{x}}_{\text{nogain}}+C_{\text{nogain}}$, to the no-gain data gives us an estimate $k_{\text{nogain}}\approx \frac{1}{f}$ (see Eq (8)). The slope of a linear fit, $y=k_{\text{gain}}{\bar{x}}_{\text{gain}}+C_{\text{gain}}$, to the data obtained with gain, given an estimate $k_{\text{gain}}\approx \frac{2g}{f}$ (see Eq (9)). Additionally, the intersections at ${\bar{x}}_{\text{gain}}=0$ and ${\bar{x}}_{\text{nogain}}=0$ gives estimates of Δ and *r*,

$$\begin{array}{c} \Delta =f\frac{C_{\text{nogain}}-C_{\text{gain}}}{2g-1},\;r^{2}=C_{\text{nogain}}-\frac{1}{12}+\frac{\Delta }{f} \end{array}$$ (10)

To estimate errors in the parameters, we use a random sub-sampling of the data into sub-sets of 1000 pixels. As the output we calculate median and interquartile ranges for each of the parameters. As an alternative to random subsampling one could use bootstrapping [21] of the original data.

### Estimating the background illumination strength, λ_*bg*_

With the camera parameters, *chipParams*, estimated once and for all, we are set to perform photophysical image analysis. To this end, we assume that the background pixels in the image at hand are described by a single Poisson parameter λ_*bg*_. For such an image, we estimate λ_*bg*_ using a procedure which makes no assumptions about the signal-pixel intensity distribution in the image.

In our method for estimating λ_*bg*_, for an image which contains both background and signal pixels, we fit a *truncated* EMCCD-PMF to the *lower part* of the image count histogram with λ_*bg*_ as a fit parameter (*chipParams* are known). The lower part of is here defined as all image counts below an optimal truncation point (threshold), $N_{icr}^{bg}$. This truncation point is chosen such that the lower part of the histogram contain image counts with an overwhelming majority coming from true background pixels. The remaining part of the histogram then contains “outliers” and consists of counts from signal and remaining (uncertain) background pixels. In order to determine $N_{icr}^{bg}$, we combine maximum likelihood estimation of λ_*bg*_ for truncated data with a *χ*^2^ goodness-of-fit tests at several different truncation points. The quantity $N_{icr}^{bg}$ is then the largest truncation point for which we obtained an acceptable fit.

The details of our procedure are:

- Sort the image counts for each pixel, thereby yielding a sorted image count list:

$S=\{n_{icr}^{\left(1\right)},n_{icr}^{\left(2\right)},\ldots ,n_{icr}^{\left(m\right)}\}$ . Here, *m* is the total number of pixels.
- Estimate λ_*bg*_ using maximum likelihood estimation applied to the truncated data for a set of truncation points (lowest is set to 25% of data). In this step, we form the joint truncated probability $\prod_{j}\frac{pmf(n_{icr}^{\left(j\right)}|\theta )}{cdf(N_{icr}^{bg}|\;\theta )}$, where the PMF is obtained by numerically evaluating the inverse Fourier transform of the characteristic function in Eq (3), the CDF is the cumulative sum over PMFs (see Sec. S1 and S2 in S1 Text) and *θ* = {*chipParams*, λ_*bg*_}. In the truncated MLE procedure, we set the initial estimate as $\lambda_{bg}=\left(n_{icr}^{\left\lfloor m/2\right\rfloor }-\Delta \right)/\left(g/f\right)$ (see Eq 4).
- Calculate the goodness-of-fit *χ*^2^ score for each of the truncation points. In this procedure we divide the data into five unique image count intervals, based on quantiles for the fitted PMF, to keep the number of degrees of freedom in the test (see below) the same for each truncation point.
- Set as $N_{icr}^{bg}$ the largest truncation point passing the *χ*^2^ test (with one estimated parameter) [22] at a significance level set by a p-value threshold *p*_*GoF*_. In here, we use *p*_*GoF*_ = 0.01.

As the output from the procedure above, we obtain an estimate for λ_*bg*_, but also obtain an intensity threshold, $N_{icr}^{bg}$, such that the truncated distribution fit is good (i.e., we are “certain” that the image counts below $N_{icr}^{bg}$ originate from background pixels).

Note that our procedure implicitly assumes that the image is acquired under uniform illumination (so that λ_*bg*_ does not vary over the image region of interest).

### Unsupervised probabilistic image thresholding

Image thresholding (binarization) is based on setting a threshold $N_{icr}^{thresh}$ for the recorded image counts. Pixels which have image counts $\leq N_{icr}^{thresh}$ are turned “black” and pixels with an image count $>N_{icr}^{thresh}$ are turned “white”. As we illustrate in S9 Fig (panels c and d) in S1 Text, two common unsupervised thresholding approaches, the Otsu method and adaptive thresholding, for automatically determining $N_{icr}^{thresh}$ can yield uncontrolled results.

The main novelty of our new thresholding method, as detailed below, is that we provide *a priori* error estimates through two conditional probabilities: *p*(black|s)—the probability that an actual signal (s) pixel is deemed black at a given intensity threshold, and *p*(white|bg)—the probability that an actual background (bg) pixel is deemed white at this threshold. In Sec. S6 in S1 Text, we relate these two quantities to commonly used statistical measures such as the false positive rate (FPR) = fraction of incorrectly classified background pixels, the false negative rate (FNR) = fraction of incorrectly classified signal pixels, the accuracy (ACC) = fraction of correctly classified pixels among all the pixels, FDR = fraction of false positives among positives and FOR = fraction of false negatives among negatives.

Our unsupervised probabilistic image thresholding method uses as input a predetermined p-value threshold, *p*_*binarize*_ (i.e. predetermined value of the “wanted” FPR in the thresholded image). To convert *p*_*binarize*_ to an intensity threshold $N_{icr}^{thresh}$, we use the full PMF for the background photons (including camera-induced noise), see previous subsection. From this PMF, we can calculate the probability that an actual background pixel (bg) would yield an image count which exceeds $N_{icr}^{thresh}$ (and hence be deemed to be a white pixel) as:

$$\begin{array}{c} p\left(\text{white}|\text{bg}\right)=1-\text{cdf}\left(N_{icr}^{thresh}|\;\theta \right). \end{array}$$ (11)

By setting the left-hand-side above = *p*_*binarize*_ and inverting, we estimate the image count threshold $N_{icr}^{thresh}$ at which the probability (p-value, *p*_*binarize*_) is no more than, say, 1%. Applying the threshold $N_{icr}^{thresh}$ onto the image at hand then yields a black and white (binarized) image, where the error rate *p*(white|bg) is known *a priori*.

Given the image binarization threshold $N_{icr}^{thresh}$, we can also estimate the other error rate, *p*(black|s). To achieve this task, we first notice that the number of background pixels *n*_*bg*_ in the image can be determined through:

$$\begin{array}{c} \left(\#\;\text{pixels}\;\text{with}\;\text{an}\;\text{image}\;\text{count}\leq N_{j}\right)=n_{\text{bg}}\text{cdf}\left(N_{j}|\;\theta \right) \end{array}$$ (12)

for $N_{j}<N_{icr}^{bg}$. Hence, by plotting (# pixels with an image count ≤ *N*_*j*_) as a function cdf(*N*_*j*_| *θ*), we extract *n*_bg_ as the slope of a fitted linear function. The total number of signal pixels is then estimated through *n*_s_ = *m* − *n*_bg_, where *m* is the total number of pixels in the image, as before. The number of black pixels among the background pixels is:

$$\begin{array}{c} n\left(\text{black}|\text{bg}\right)=n_{bg}\text{cdf}\left(N_{icr}^{thresh}|\;\theta \right) \end{array}$$ (13)

The number of black pixels in the binarized image is $n_{\text{black}}=\left(\#\;\text{pixels}\;\text{with}\;\text{an}\;\text{image}\;\text{count}\leq N_{icr}^{thresh}\right)$ and hence the number of black pixels among the signal pixels is

$$\begin{array}{c} n\left(\text{black}|\text{s}\right)=n_{\text{black}}-n\left(\text{black}|\text{bg}\right) \end{array}$$ (14)

As a consequence, we finally arrive at the probability that a signal pixel is deemed black in the binarized image:

$$\begin{array}{c} p\left(\text{black}|\text{s}\right)=\frac{n(\text{black}|\text{s})+1}{n_{\text{s}}+2} \end{array}$$ (15)

The +1 and +2 terms in the numerators and denominators above are introduced in order to avoid zero probabilities [23].

### Image segmentation

The goal of the image segmentation is to identify “objects” (in here, fluorescently-labeled lung cancer cell nuclei or fluorescent beads) in an image. These objects often correspond to contiguous regions of pixels, which one seeks to identify and to classify as *signal* (s) or *background* (bg) regions. In here, to keep things at a general-purpose level, by an “object” we refer to a single cell/bead, or to a cluster of touching cells/beads (separating touching objects would this would be a post-processing step which will require knowledge of the geometry of the object).

Let us now, to illustrate how our image binarization result can potentially be used in downstream image analysis tasks, introduce a heuristic image segmentation method. This is not a main novelty of this study, but included in our software package as an example application.

Our unsupervised image segmentation method assumes that erroneously classified pixels in our binarized image (see above) have a short-range spatial correlation. This in effect forces false background and false signal regions to be “small”. Our method makes use of one parameter, allowedGapLength (set = 1 in all examples) and is:

1. Find connected components of white pixels in the thresholded image (white regions) and connected components of black pixels (black regions).
2. Flip all the pixels in the white regions smaller than *w*_white_ and flip all the pixels in black regions smaller than *w*_black_ from the binarized image. By “flip” we refer to the operation of turning a white pixel black, or turning a black pixel white. Our procedure for obtaining the two thresholds, *w*_black_ and *w*_white_, is described below.
3. For each region, we calculate a p-value, *p*_seg_, based on the sum of image counts for all the pixels in a region, see Sec. S1 in S1 Text for the characteristic function used to estimate the p-values for each of the regions. This p-value serves as a quality control of the segmentation.

To determine *w*_black_ in step 2 above we find all black regions and sort these according to size (with the smallest regions size first). We then proceed through the sorted list and stop when the gap between the present region size (*w*_black_) and the next region size exceeds allowedGapLength. The procedure for determining *w*_white_ is analogous. For the case when there is no gap larger than allowedGapLength we keep no connected components/objects.

Note that our method makes no assumption about the geometry or topology of the “objects” in the image. For instance, in case the objects would have “doughnut” shapes, our method should be able to detect the holes in these objects (however, we do not have any such objects in our examples in Results).

## Results

We here demonstrate our PIA method for unsupervised probabilistic image thresholding gives *a priori* estimates for misclassification rates which are close to ground truth values (Fig 2). Prior to this we determine the camera chip parameters using a mean-variance (MV) test and estimate the background Poisson parameter directly for the image at hand (which contains both signal and background image counts). We end the Results section by showing image segmentation results using our heuristic general-purpose method. We apply our methods to both synthetic (simulated) and experimental fluorescence microscopy images.

### Estimating the camera chip parameters using calibration experiments

As a pre-processing step we need to determine the four camera chip parameters, gain *g*, analogue-to-digital unit conversion factor *f*, read-out noise parameter *r*, and camera offset (bias) parameter Δ (unless these are known from a previous calibration). To this end, we perform linear fits of the variance in image counts as a function of mean image counts for three different gain settings (see Methods for details). This procedure yields parameter estimates (median and the interquartile range) as reported in Table 1. Note that the accuracy (here quantified by the interquartile ranges) of the estimates can be improved by using a larger set of calibration experiment at different illumination intensities, or using an MLE approach [16]. It is, however, not a main aim of this study to provide a new method for estimation of the camera parameters, and we therefore settled at the current accuracy. Note that the data from differing gain settings yield consistent estimates of *r* and Δ, as it should. Since the chip parameters, except the gain, are static properties of the chip, these experiments only need to be performed once.

**Table 1:** Estimates of the chip parameters for an EMCCD camera at different gain settings. The calibration data shown in S1 Fig in S1 Text was used, and the chip parameters were calculated using Eqs (4)–(7) in Materials and Methods.

Gain	f (interquartile range)	*g*	*r*	Δ
0	35.17 (33.46–36.84)	-	-	-
50	-	11.54 (10.98–12.13)	1.45 (1.44–1.47)	27.03 (25.31–28.80)
100	-	18.82 (17.88–19.73)	1.45 (1.43–1.46)	26.37 (24.57–28.21)
300	-	46.44 (44.22–48.71)	1.46 (1.44–1.47)	27.17 (25.42–29.25)

### Estimating the Poisson parameter for the background

**Fig 1:**
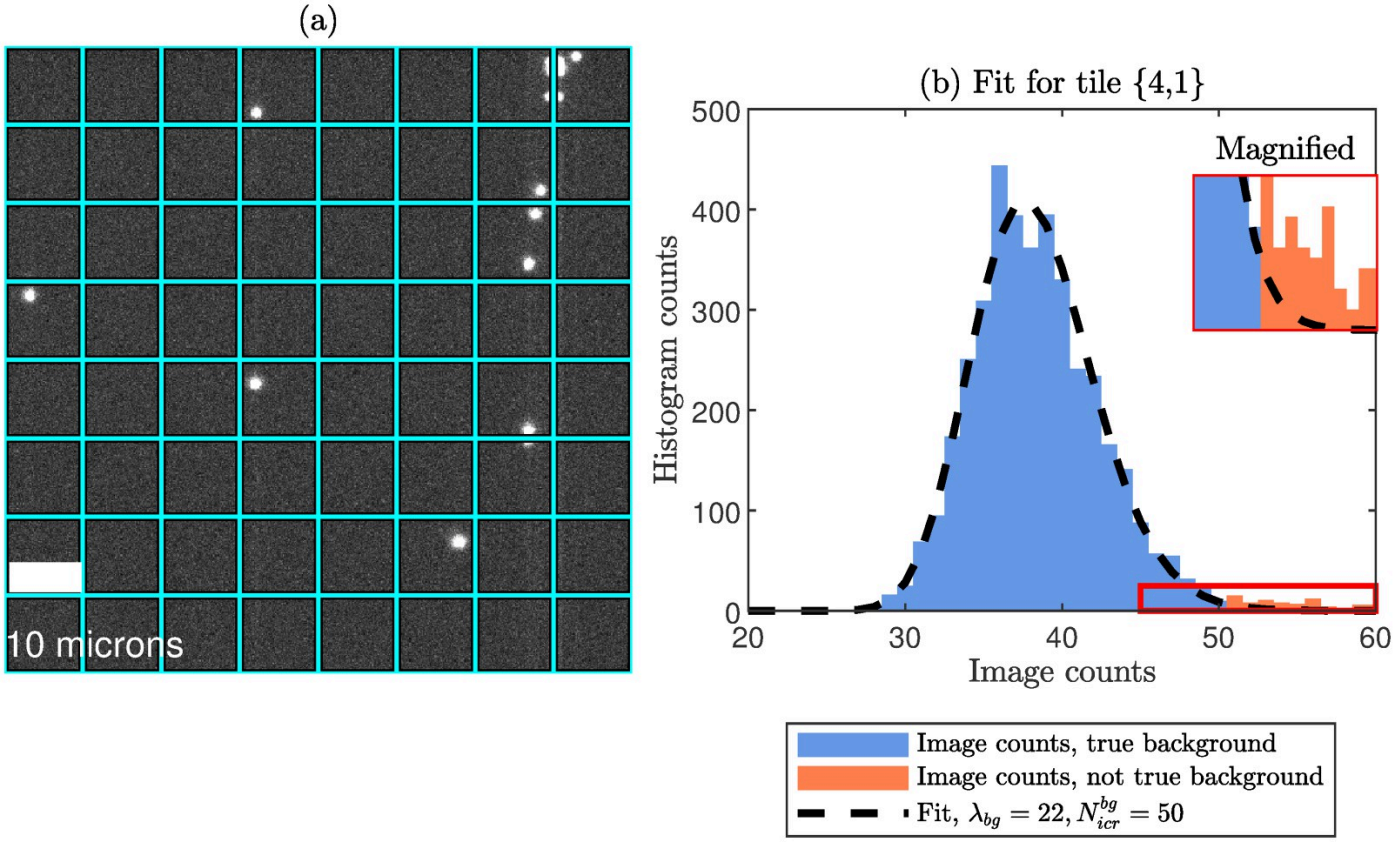
Estimating λ_*bg*_ for an image which contains both background and signal. (a) Experimental fluorescence microscopy image of fluorescent beads. Here, the image is split into tiles of size 64x64 pixels, where each tile is given a label {*row*, *column*}, where in this example *row*, *column* = 1, …, 8. The contrast is set to better display the background noise and glass slide artifacts. (b) Estimating λ_*bg*_: A histogram of the image counts for a single tile, here tile {4, 1}. The blue bars represent pixels regarded as true background, while the orange bars represent the outliers (not true background or signal pixels). The image counts threshold, $N_{icr}^{bg}=50$, separating the blue and orange bars was determined using a p-value threshold, *p*_*GoF*_ = 0.01, for the goodness-of-fit tests. The dashed black curve shows the fitted PMF for the estimated λ_*bg*_, extended to the full range of image counts (in our method, we fit a truncated PMF to the blue bars). In S5 Fig in S1 Text, we provide the estimates of λ_*bg*_ and $N_{icr}^{bg}$ for all tiles in panel a). In S8 Fig in S1 Text, we also provide two more examples of image count histograms with overlaid fits. Examples of fits to histograms for synthetic images at varying SNR is found in Sec. S7 in S1 Text. A major novelty of our method is that we are able to estimate λ_*bg*_ for a arbitrary fluorescence image even though the image contains signal pixels.

The results obtained from our novel method for estimating the Poisson parameter of the background (λ_*bg*_) directly from an input image which contains both background and signal is shown in Fig 1. As input we use an experimental image of 250 nm diameter fluorescent polystyrene beads adsorbed onto a surface, where the image was split into several tiles. Panel a) shows an example of such an image acquired with the settings labeled “Gain 100” in Table 1. In panel b), we show image count histogram for one of the tiles in panel a). In order to estimate λ_*bg*_ from this histogram, we fit a *truncated* PMF to the data at several different truncation points. For each truncation point, we run a goodness-of-fit test and choose the optimal threshold, $N_{icr}^{bg}$, as the largest truncation point which passed the tests, see Methods for details. Our estimate of λ_*bg*_ is the value obtained by the fit to the data at a threshold $N_{icr}^{bg}$. In the histogram, blue bars represent intensities below $N_{icr}^{bg}$ (“certain” background pixels). The orange bars represent the outliers, i.e. image count values which are not certain to be background (remaining background or signal pixels). Our procedure yields an estimate λ_*bg*_ = 22, i.e., in this particular tile on average 22 photons are incident on each background pixel during the exposure time (assuming quantum efficiency = 1, and neglecting the spurious charge, see Methods). Notice that our novel procedure automatically makes estimates of the statistical properties of the background, although the image contains both background and signal pixels. The fitted EMCCD-PMF, extended to the full image count range, is given by the black curve in Fig 1(b).

The reason that we split the image into tiles is that the illumination intensity varies slightly over the image at hand, thus violating the model assumptions from Methods. By tiling the image we get roughly constant illumination over each tile, thereby making sure that the model assumptions are satisfied and, as a consequence, most of the tiles pass the goodness-of-fit p-value threshold, see S5 Fig in S1 Text. We leave it as a future challenge to extend our approach to situations of strong non-uniform illumination.

In the experimental image in Fig 1, the vast majority of the pixels are background pixels and the signal-to-noise ratio is rather high. To show that our procedure works also for smaller SNR, S4 Fig in S1 Text show examples of fits to histograms for synthetic images at varying SNR, where we demonstrate that our procedure for estimating λ_*bg*_ works also for smaller SNRs.

### Unsupervised probabilistic image thresholding

The estimates of the camera parameters and our automatically determined λ_*bg*_ allow us to perform unsupervised probabilistic image thresholding, as described in Methods. To quantitatively test out our image thresholding approach (see Methods), we apply it to synthetically generated images of fluorescent beads. Our procedure for generating such images is described in Sec. S4 in S1 Text. The SNR in these images is defined [24], such that $\text{SNR}=\lambda_{sig}/\left(\sqrt{\lambda_{sig}+\lambda_{bg}}\right)$ where λ_*sig*_ is the Poisson parameter for a signal pixel. Since we use synthetic images, we know the ground truth, i.e., which pixel is a background pixel and which is a signal pixel, and can use this information to estimate the quality of the thresholding. To this end, we use five observables: FPR, FNR, ACC, FDR, FOR. The main novelty of our method is that we can estimate all of these rates *a priori*, i.e., even if the ground truth is not known.

**Fig 2:**
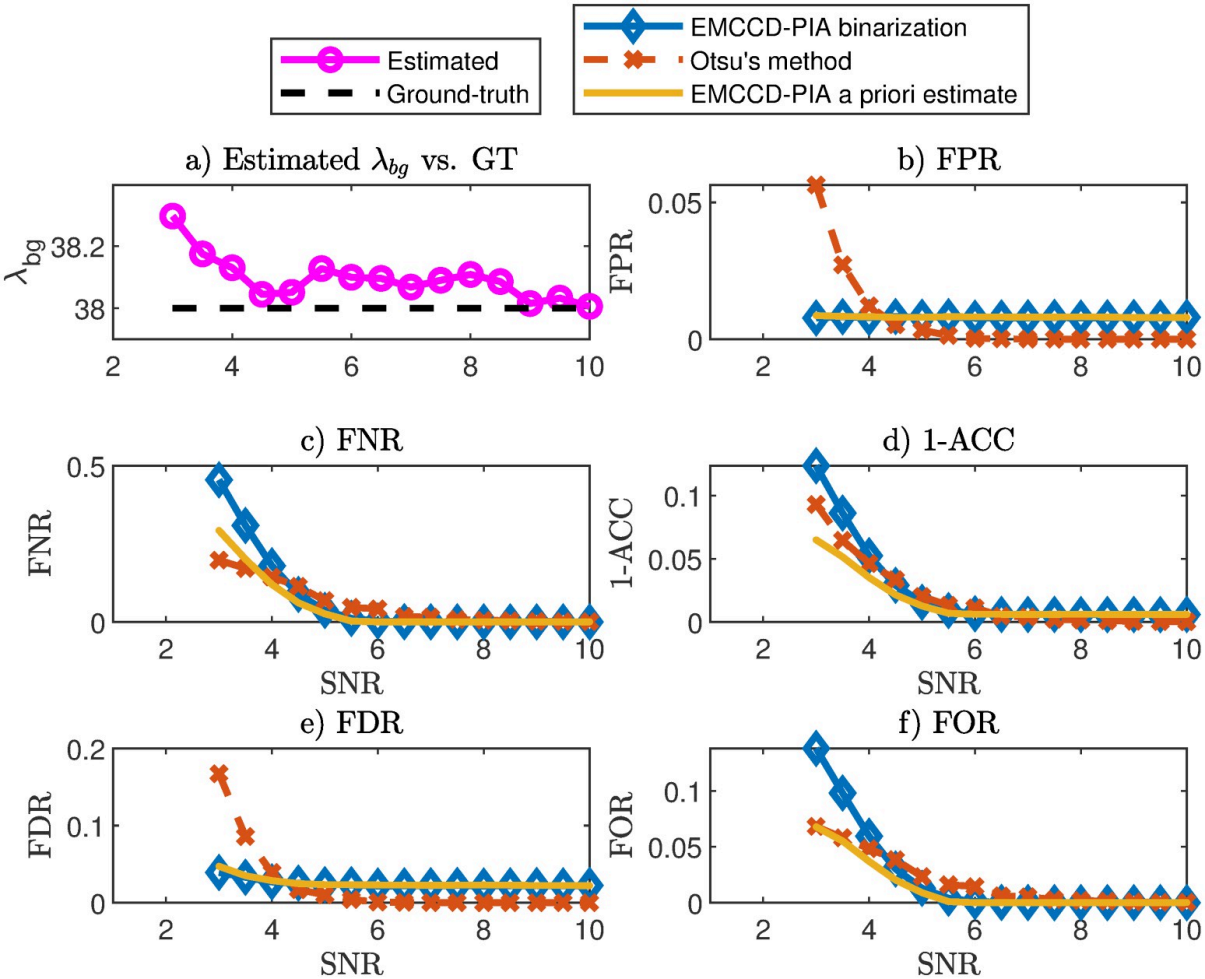
Photophysical image binarization. Synthetic images of fluorescent beads were generated as described in Sec. S4 in S1 Text at different signal-to-noise ratios (with known values of λ_*bg*_ and λ_*sig*_). For these images we know the ground truth pixel identity, i.e., which pixels are background and which are signal. (a) Estimated λ_*bg*_ compared to the ground truth value. (b-f) Our image binarization method was applied to the synthetic images (with binarization threshold *p*_binarize_). The result was then compared to ground truth pixel identity, with success rates quantified through five statistical observables (blue). The orange marks correspond to our *a priori* prediction of the same observables (obtained without the knowledge of the ground truth). We also show Otsu’s method compared to ground truth pixel identities (red). In our method we have excellent control over the FPR, i.e., the fraction of white pixels in background regions. We also have good control over the FNR. Particularly important to notice is that our *a priori* prediction (orange) for all statistical observables agree very well with ground truths (blue). This agreement is the strength of our novel thresholding method, which hence opens up for unsupervised image thresholding where the error for the classification for each pixel is obtained *a priori*. The image count threshold for the binarization was determined through a p-value threshold *p*_binarize_ = 0.01.

Our thresholding (binarization) method uses a p-value threshold, *p*_binarize_ (not to be confused with the p-value threshold used in the goodness-of-fit test, *p*_*GoF*_) which controls the FPR. The results for the FPR, FNR, 1-ACC, FDR and FOR are shown in Fig 2 where we compare to our *a priori* predictions (see Methods) and to the Otsu method. Notice that with our probabilistic thresholding method, we have excellent control over the FPR and, unlike the Otsu method, can predict the other statistical observables *a priori*. The details of our procedure for *a priori* estimation of the FPR, FNR, 1-ACC, FDR and FOR is described in the Methods section and in Sec. S6 in S1 Text. The success of our unsupervised probabilistic thresholding method is quantified by the good agreement between the blue marks (binarization at a threshold set by *p*_binarize_ vs ground truth) and the orange curves (*a priori* estimates).

In S9 Fig in S1 Text, we applied our image thresholding method to the experimental data from Fig 1. Compared to two common unsupervised thresholding approaches, the Otsu method and adaptive thresholding, it is visually clear (no ground truth is here available) that our method gives good control over the FPR.

### Image segmentation

**Fig 3:**
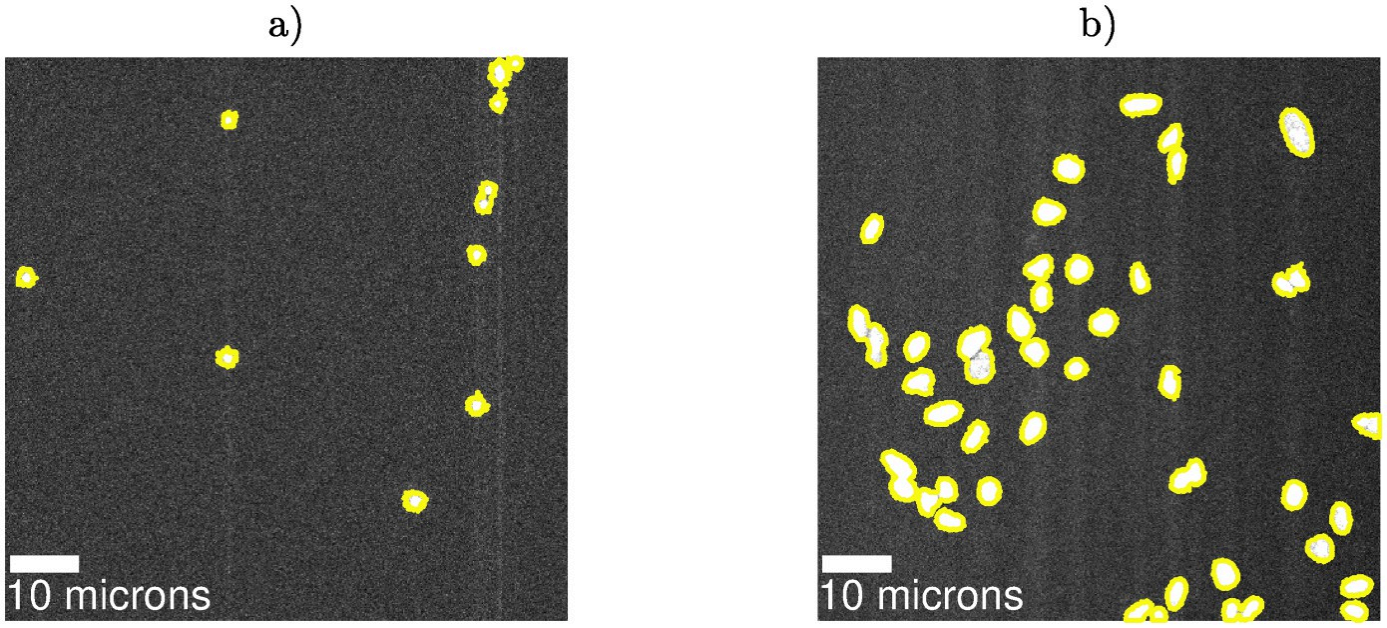
Photophysical image segmentation. (a) Beads image with detected regions (connected components of white pixels). The original image can be found in Fig 1a). The yellow pixels are boundary points to the detection regions. The binarized image used as input to our segmentation approach is found in S9 Fig (panel b) in S1 Text (b) Lung cell nuclei image with detected regions. The original image can be found in S7 Fig in S1 Text (a). Segmenting larger object with lower magnification is more challenging, since the background illumination will vary. We here tile the image and set p-value thresholds *p*_*GoF*_ = 0.01 and *p*_*binarize*_ = 0.01 and region size gap threshold = 1. An example of the associated image count histogram with an overlaid fit is found in S7 Fig in S1 Text. Notice that our image segmentation method performs visually well on both experiments.

The first application of our unsupervised image segmentation method is the image in Fig 1. The result is shown in Fig 3(a), where we see that, visually (no ground truth is available here), our method performs very well. Our segmentation method uses the binarized image in S9 Fig (panel b) in S1 Text as input. For the image in Fig 3(a), the p-values for the regions, *p*_*seg*_, were all below 0.01.

We next apply our image segmentation framework to more challenging images, namely images of stained lung adenocarcinoma A549 cell nuclei. These images are taken with a lower magnification (20 X) and the gain setting “Gain 100” in Table 1. At this magnification, the illumination varies across the field of view, violating our assumption of constant background. In addition, the non-uniform staining of such cell nuclei, as well as their varying shapes and sizes, should make the image harder to segment. Despite these challenges, our method, after tiling of the image (see Materials and methods), visually performs very well, as the segmentation results shown in Fig 3(b) illustrate.

To illustrate that our method is insensitive to the geometry of the objects, S10 Fig in S1 Text shows segmentation results for a synthetic image with objects of more complicated shapes.

## Summary and discussion

In this study, we introduced EMCCD Photophysical Image Analysis methods and tools which make full use of the physical knowledge of photon statistics and the physical mechanisms underlying EMCCD cameras. Our image thresholding method is unsupervised (no training data set required) and probabilistic (number of wrongly classified pixels can be estimated *a priori*).

Any image originating from fluorescence imaging technologies, will contain both signal and background. The signal consists of the photons from the dyes of interest. The background can have several origins. For instance, ambient light may leak into the microscope and reach the detector. Autofluorescence from the sample as well as from the optical setup is a common problem when working with short excitation wavelengths. Optical filters are not perfect and may transmit unwanted light. However, as long as all these background photon sources follow stationary Poisson statistics, our PIA pipeline applies. This fortunate fact follows from the fact that the sum of random numbers drawn from different Poisson distributions (with parameter values λ_*i*_, *i* = 1, …, number source types) follow another Poisson distribution (with parameter λ_*bg*_ = ∑_*i*_ λ_*i*_). Note that within our PIA methods, we make no particular assumption about the statistics for the signal pixels. The signal can, for instance, be time-varying, spatially varying, or be described by image count histograms which are multimodal, etc.

A few future challenges remain. As already mentioned, the assumption of uniform background illumination is sometimes violated, which, for stronger gradients in the illumination, requires adaption of our procedure for estimating λ_*bg*_ using a model also for the illumination profile. A second future challenge is to deal with the fact that the gain factor cannot be expected to be perfectly uniform across the range of pixels. This may lead to a broadening of the distribution under certain conditions, which we, however, have not observed.

To increase the utility of our method further, the framework should be generalized to handle images acquired via complementary metal-oxide-semiconductor (CMOS) [25] type cameras. The theoretical challenge is that the response function for CMOS cameras differ from the one presented in Methods. We plan to tackle this challenge in a future study.

Even though our application in this study have been on fluorescence images, we expect variants of our methodology to apply to other types of optical imaging technologies where control over the error rates in classification are important. In particular, we expect our approach, with appropriate modifications for each application, to be useful especially in those cases where image quality is poor (cellphone microscopy, USB microscopy) [26] or where the photon budget is tight (various super resolution microscopies, especially STED). Furthermore, there might be microscopies where fluctuations associated with the staining can be leveraged, for example STORM/FPALM with blinking statistics and/or photoswitching statistics contributing, as well as PAINT where the binding and unbinding of dye molecules contribute to the fluctuations in the signal.

Since our image analysis pipeline is fast and unsupervised (no training required), it is ideal for optical imaging technologies in high-throughput settings [2–4]. Unlike many other image analysis tools, our method does not include any parameters which need to be manually adjusted by the user and hence opens up for full reproducibility in image analysis where no manual decisions need to be made. In addition, our method is probabilistic, and allows *a priori* estimation of the expected number of wrongly classified pixels, which, in turn, is crucial information for downstream error analysis and corrections.

Image analysis is a scientific discipline traditionally considered as a branch of mathematics. We hope this study will inspire further work in the field of *photophysical image analysis*. In this field, we seek to make full use of physical modeling to develop automated, unsupervised methods and provide *a priori* error rates for different image analysis tasks.

## Supporting information

S1 Text Detailed description of our methods and supporting figures. We here provide all necessary details of our PIA methods and supporting figures.
(PDF)
