# Supplementary material for: Photophysical image analysis: Unsupervised probabilistic thresholding for images from electron-multiplying charge-coupled devices
Source: PLoS One. 2024 Apr 5;19(4):e0300122. doi: 10.1371/journal.pone.0300122 (PMC10997106; doi:10.1371/journal.pone.0300122)
Supplement: S1 Text — We here provide all necessary details of our PIA methods and supporting figures. (PDF) [file pone.0300122.s001.pdf]

Supplementary information – Photophysical image analysis: unsupervised probabilistic thresholding for images from electron-multiplying charge-coupled devices

Jens Krog[1◑], Albertas Dvirnas[1◑], Oskar E. Ström[2], Jason P. Beech[2], Jonas O. Tegenfeldt[2], Vilhelm Müller[3], Fredrik Westerlund[3], Tobias Ambjörnsson[1*]

1 Centre for Environmental and Climate Science, Lund University, Sweden
2 Department of Physics and NanoLund, Lund University, Lund, Sweden
3 Department of Life Sciences, Chalmers University of Technology, Gothenburg, Sweden

◑These authors contributed equally to this work.
* tobias.ambjornsson@cec.lu.se

S1: Derivation of the characteristic function for the image counts

In here we derive our new expression for the characteristic function for the image counts recorded by an EMCCD camera. In our derivation, the incoming number of electrons, n_{ie} and the rounded image counts, n_{icr} are discrete-values quantities, whereas the number of outgoing electrons, n_{oe} and the image counts, n_{ic}, are treated as continuum-valued variables.

The distribution for the incoming electrons n_{ie} is

$$p(n_{ie}|\lambda) = \frac{\lambda^{n_{ie}}}{n_{ie}!} e^{-\lambda}, \tag{S.1}$$

where we have the normalization condition: $\sum_{n_{ie}=0}^{\infty} p(n_{ie}|\lambda) = 1$.

The conditionals for the outgoing electrons n_{oe} with gain g is given in Eq. (1) in the main text, where we have a normalization condition: $\int_0^{\infty} p(n_{oe}|g,\lambda) dn_{oe} = 1$. We may marginalize out the parameter n_{ie} such that

$$p(n_{oe}|g,\lambda) = \sum_{n_{ie}=0}^{\infty} p(n_{oe}|n_{ie},g) p(n_{ie}|\lambda). \tag{S.2}$$

We then recover a simple form of the characteristic function for n_{oe}:

$$\langle e^{ipn_{oe}} \rangle = \int_0^{\infty} e^{ipn_{oe}} p(n_{oe}|g,\lambda) dn_{oe} = \int_0^{\infty} e^{ipn_{oe}} \left(\delta(n_{oe}) + \sum_{n_{ie}=1}^{\infty} \frac{n_{oe}^{n_{ie}-1} e^{-n_{oe}/g}}{\Gamma(n_{ie}) g^{n_{ie}}} \frac{\lambda^{n_{ie}}}{n_{ie}!} \right) e^{-\lambda} dn_{oe} \tag{S.3}$$

$$= e^{-\lambda} + \sum_{n_{ie}=1}^{\infty} \left(\frac{\lambda}{1-ipg} \right)^{n_{ie}} \frac{e^{-\lambda}}{n_{ie}!} = \exp\left[\lambda \left(\frac{1}{1-ipg} - 1 \right) \right], \tag{S.4}$$

where we switched the order of the integral and the sum, used the fact that characteristic function of the Dirac delta function is equal to 1 and the definition of the Gamma function.

Similarly, since the number of output electrons n_{oe} is a hidden parameter, we again marginalize to obtain

$$p(n_{ic}|f,r,\Delta,g,\lambda) = \int_0^{\infty} p(n_{ic}|n_{oe},f,r,\Delta) p(n_{oe}|g,\lambda) dn_{oe}, \tag{S.5}$$

where $p(n_{ic}|n_{oe},f,r,\Delta)$ is defined in Eq. (2) in the main text. The characteristic function for the image count n_{ic} is then:

$$\langle e^{ipn_{ic}} \rangle = \int_0^{\infty} e^{ipn_{ic}} p(n_{ic}|f,r,\Delta,g,\lambda) dn_{ic} = \int_0^{\infty} e^{ipn_{ic}} \left(\int_0^{\infty} \frac{1}{\sqrt{2\pi r^2}} e^{-\frac{(n_{ic}-n_{oe}/f-\Delta)^2}{2r^2}} p(n_{oe}|g,\lambda) dn_{oe} \right) dn_{ic} \tag{S.6}$$

$$\approx \int_0^{\infty} p(n_{oe}|g,\lambda) e^{ip(n_{oe}/f+\Delta)} \left(\int_{-\infty}^{\infty} \frac{1}{\sqrt{2\pi\sigma^2}} e^{-\frac{\left(n_{ic}-\frac{n_{oe}}{f}-\Delta\right)^2}{2r^2}} e^{ip(n_{ic}-n_{oe}/f-\Delta)} \mathrm{dn_{ic}} \right) dn_{oe} \tag{S.7}$$

$$= e^{ip\Delta} \langle e^{i(p/f)\, n_{oe}} \rangle \exp\left[-\frac{p^2 r^2}{2} \right] = \exp\left[-\frac{p^2 r^2}{2} + ip\Delta \right] \exp\left[\lambda \left(\frac{1}{1-ip(g/f)} - 1 \right) \right], \tag{S.8}$$

which is just the product of the characteristic function for n_{oe} and characteristic function for Gaussian distribution of mean Δ and standard deviation r. On the second line above, we changed the lower integration limit over the integral over n_{ic} to minus infinity, which is justified if Δ is large.

Finally, to statistically include the effect of rounding, we approximate the rounded image counts, n_{icr}, by adding a uniformly distributed random number between $-1/2$ and $1/2$ to n_{ic}. The characteristic function for n_{icr} is then obtained by multiplying $\langle e^{ipn_{ic}}\rangle$ by the characteristic function of the uniform distribution , i.e.

$$\langle e^{ipn_{icr}}\rangle \simeq \exp\left[-\frac{p^2r^2}{2}+ip\Delta\right]\exp\left[\lambda\left(\frac{1}{1-ip(g/f)}-1\right)\right]\frac{\sin(p/2)}{p/2}. \tag{S.9}$$

Numerical Fourier-inversion of this CF give the image count PMF and CDF (see Sec. S2).

If instead the electron multiplication is turned off, an identical calculation, but now using the associated expression for $p(n_{oe}|n_{ie},g)$ for the no gain case (see Eq. (1) in the main text), yields:

$$\langle e^{ipn_{icr}}\rangle_{\text{nogain}} = \exp\left[-\frac{p^2r^2}{2}+ip\Delta\right]\exp\left[\lambda(e^{ip/f}-1)\right]\frac{\sin(p/2)}{p/2}. \tag{S.10}$$

As a consistency check of our image segmentation method, we calculate p-values for the summed image counts in each detected region. This calculation requires the PMF and CDF for summed image counts. Therefore, let us consider the summed image counts from m independent pixels, $N=\sum_{j=1}^{m} n_{icr}^{(j)}$. Since the characteristic function for the sum of independent random numbers is the product of the individual characteristic functions, we have:

$$\langle e^{ipN}\rangle \simeq \exp\left[-\frac{p^2mr^2}{2}+ipm\Delta\right]\exp\left[m\lambda\left(\frac{1}{1-ip(g/f)}-1\right)\right]\left(\frac{\sin(p/2)}{p/2}\right)^m \tag{S.11}$$

and

$$\langle e^{ipN}\rangle_{\text{nogain}} = \exp\left[-\frac{p^2mr^2}{2}+ipm\Delta\right]\exp\left[m\lambda(e^{ip/f}-1)\right]\left(\frac{\sin(p/2)}{p/2}\right)^m. \tag{S.12}$$

Numerical inversion of these CFs give the required PMF and CDF (see Sec. S2).

S2: Inversion of Characteristic function

The Fourier-inverse of the characteristic function is based on the approach in [3] (see also [4]) . We use the Gil-Pelaez inversion formula [5,6] to calculate PMF from the characteristic function $\langle e^{ipn_{icr}}\rangle$:

$$\text{pmf}_Y(n_{icr}|\ \theta) = \frac{1}{\pi}\int_0^{\pi}\Re\left(e^{-ipy}\langle e^{ipn_{icr}}\rangle\right)dp \tag{S.13}$$

where $\Re(f)$ denotes the real part of f. Here the integral is taken up to π since the underlying random variable Y is discrete. We approximate Eq. (S.13) by using a trapezoidal quadrature to get

$$\text{pmf}_Y(n_{icr}|\ \theta) \approx \frac{\delta_p}{\pi}\sum_{j=0}^{N} w_j\Re\left(e^{-ip_jy}\langle e^{ip_jn_{icr}}\rangle\right) \tag{S.14}$$

Where $\delta_p=\frac{2\pi}{U-L}$, $N=\frac{U-L}{2}$ and $p_j=j\delta_p$ with $j=0,\ldots,N$. Here, the interval (L,U) specifies the range for the values of n_{icr}, here estimated to be

$$L=\mu-6\sigma^2,\quad U=\mu+6\sigma^2 \tag{S.15}$$

where μ and σ are from Eqs. (4) and (5) in the main text.

Eq. (S.13) is evaluated for positive integers, and the associated cumulative distribution function (CDF) is then obtained through:

$$\text{cdf}_Y(n_{icr}|\ \theta) = \sum_{n=1}^{n_{icr}}\text{pmf}_Y(n|\ \theta) \tag{S.16}$$

S3: Experimental details

All images were taken with an inverted Nikon Eclipse Ti microscope (Nikon Corporation, Tokyo, Japan), an Andor iXon DU-897 EMCCD camera (Andor Technology, Belfast, Northern Ireland) and Lumencor SOLA light engine (Lumencor Inc, OR, USA). The following Nikon objectives were used: 100x oil immersion (NA=1.4, Plan Apo VC), 20x air (NA=0.75, Plan Apo λ OFN25). FITC filter cube (EX 482/35 DM 505, EM 536/40) was used for the beads and DAPI filter cube (EX 387/11, DM, EM 447/60) was used for the lung cancer cells. The camera exposure time was 10 ms for all images and images in a video stack were captured with 13.76 frames per second.

Fluorescent beads (250 nm diameter green polystyrene, CV of 5%, Thermo Fisher Scientific, MA, USA) were diluted 100 times in purified water (Milli-Q) and pipetted on-to a standard glass slide (76x26 mm, soda-lime, Menzel Gläser, Thermo Fisher Scientific). The dispersion was sealed with a coverslip (No. 1.5H coverslip, Marienfeld, Lauda-Königshofen, Germany) and nail polish to prevent evaporation.

Human lung adenocarcinoma A549 cells (ECCC, distributor Sigma Aldrich, MO, USA) were cultured in Ham's F12K media (Gibco), fixated under paraformaldehyde and then stained with Hoechst33342 (Sigma Aldrich) and AlexaFluor 488 Phalloidin (Thermo Fisher Scientific) which stains the cytoskeleton and is not imaged here. More detailed descriptions of the sample preparation can be found in the study by Abariute et al. [1]

S4: Synthetic images

The synthetic images used to test the performance of our segmentation method were generated by first placing beads with a fixed radius (10 pixels for 100% zoom, i.e. around 1 micron) at random positions in an image. To make sure that the ground truths were well-defined, no optical smearing was added. The associated black and white image was then transformed by adding background noise to generate a photon image following the EMCCD noise model [2] and using the noise model chipParams parameters for the "Gain 100" in Table 1 in the main text. In practice, we calculate Poisson-Gamma distributed random numbers for each pixel (if pixel is a signal pixel, then we use $\lambda_{\text{sig}} + \lambda_{\text{bg}}$ as Poisson distribution parameter, and λ_{bg} for background pixel), and then add normally-distributed "read-out" noise with mean Δ and variance r^2. Finally, the image counts are rounded to the nearest integer. The parameter λ_{sig} was chosen based on the required signal-to-noise ratio (which ranged from 3 to 10) and derived from the SNR formula introduced in the main text, which gives

$$\lambda_{\text{sig}} = \text{SNR} \cdot \frac{\text{SNR} + \sqrt{\text{SNR}^2 + 4 \cdot \lambda_{\text{bg}}}}{2}; \tag{S.17}$$

S5: Determining the chip parameters for synthetic and experimental calibration data

In Fig. S1 we show results of our method for determining an estimate of the gain g and the analog-to-digital conversion factor for three different gain settings (as taken from the settings of the camera), 50, 100, and 300. In Fig. S2 we show

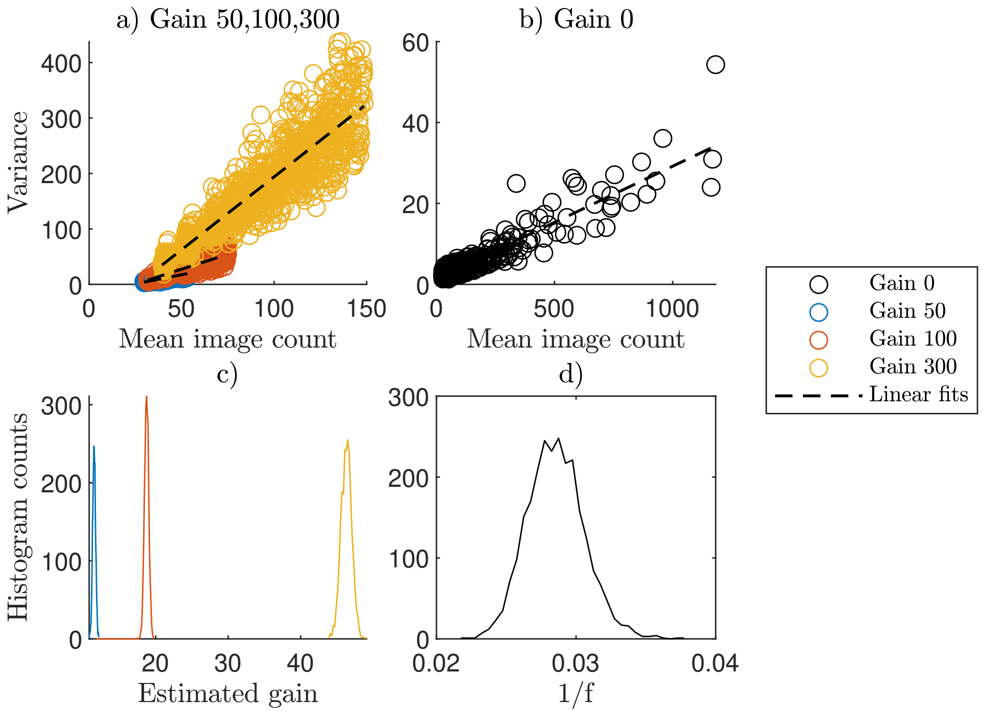

Fig S1. Determining the chip parameters through a set of calibration experiments. (a-b) The mean and variance for pixels randomly drawn from experimental image stacks is shown for the four different gain settings from the camera (0, 50, 100, and 300). The dashed lines represent the linear fits used to calculate the parameter estimates given in Table 1 in the main text. We made 2500 draws in total and each draw had 1000 pixels. (c-d) Distributions of estimated (fitted) gain parameters g as well as 1/f. Notice that the estimated parameters have rather narrow and symmetric distributions.

the estimated chip parameters for synthetic data. Notice the similarity of Figures S1 and S2.

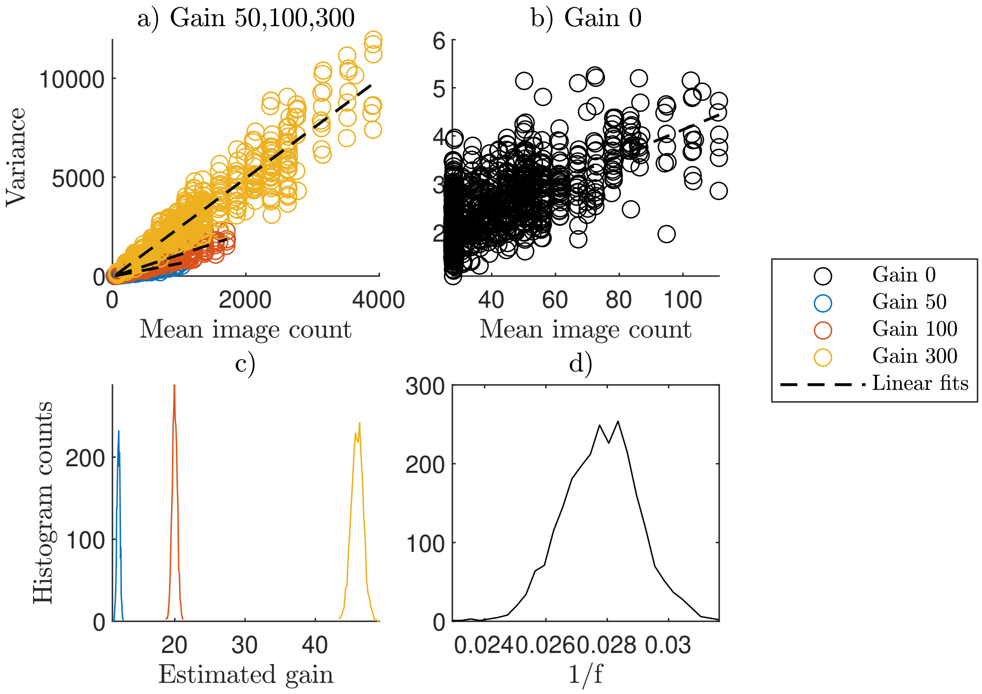

Fig S2. Estimating the chip parameters using synthetic calibration data. (Top) The mean and variance for pixels randomly drawn from synthetic image stacks is shown for the four different gain settings from the camera (0, 50, 100, and 300). Our procedure for generating synthetic images is described in Sec. S4. The dashed lines are the linear fits used to calculate the parameter estimates with a procedure described in the main text. We made 2500 draws in total and each draw had 1000 pixels. (Bottom) Distributions of estimated (fitted) gain parameters g as well as the inverse analog-to-digital conversion factor. Notice that the parameter distribution obtained from the synthetic images are similar to the distribution obtained from real images, compare to Figure 1 in the main text.

S6: Definition of statistical quantities for validation

In here we define statistical observables used in our photophysical image analysis pipeline and also utilized for quantifying the accuracy of our pipeline.

Whenever a threshold is imposed on an image count histogram there will be errors. To quantify these errors, we refer to a black pixel (given some intensity threshold) as a ”negative” and a white pixel as a ”positive”. There will then be four types of pixels

(i) true negatives (TN) = background pixels which are correctly classified as black pixels;

(ii) false negatives (FN) = signal pixels which are classified as black;

(iii) true positives (TP) = signal pixels which are correctly classified as white;

(iv) false positives (FP) = background pixels which are classified as white,

see also Table S1.

Estimated/Ground truth	background	signal
black	true negative (TN)	False negative (FN)
white	false positive (FP)	true positive (TP)

Table S1. Notation used when classifying pixels.

The difference between the four types of pixels are illustrated in Fig. S3. Note that the classification of individual pixels according to the four labels require us to have ground truths available for all pixels. In the absence of ground truth,

we can, however, still make statistical predictions on the error in classifications through the method introduced in the main text.

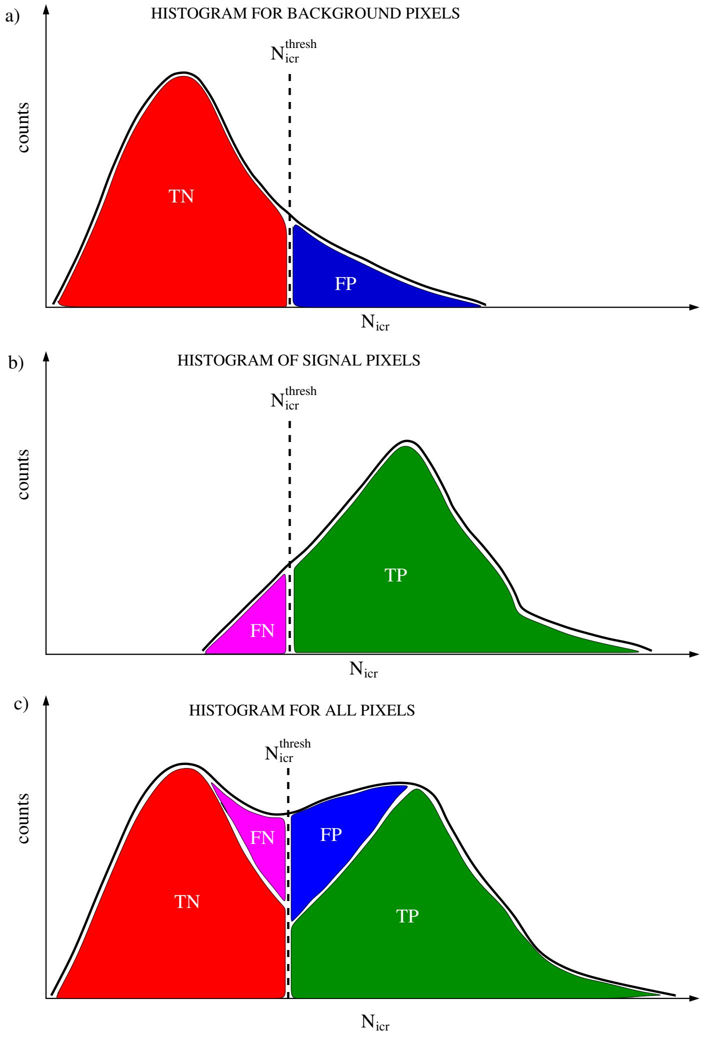

Fig S3. Definition of true negatives (TN), false negatives (FN), false positives (FP) and true positives (TP). An image in general consists of both signal pixels (positives) and background pixels (negatives). By applying a threshold N_{icr}^{thresh} to the image counts N_{icr} for each pixel one seeks to recover the signal and background pixels. However, in this process one typically makes errors, leading to FPs and FNs. a) Schematic illustration of image counts for the background pixels in an image. b) Schematic illustration of the image counts for signal pixels in an image. c) Schematic illustration of the image counts in an image which contains both background and signal pixels.

Based on the TN, FN, TP and FP, a number of statistical measures are commonly used. We here relate these such observables to the conditional probabilities: $p(\text{white}|\text{bg})$ and $p(\text{black}|\text{s})$ introduced in the main text. Notice that we have the normalization conditions: $p(\text{black}|\text{bg}) + p(\text{white}|\text{bg}) = 1$ and $p(\text{black}|\text{s}) + p(\text{white}|\text{s}) = 1$. We have

$$\begin{aligned}
\text{FPR} &= \text{false positive rate} = \frac{\text{FP}}{\text{FP+TN}} = p(\text{white}|\text{bg}) \\
\text{TNR} &= \text{true negative rate} = 1 - \text{FPR} = p(\text{black}|\text{bg}) \\
\text{FNR} &= \text{false negative rate} = \frac{\text{FN}}{\text{FN+TP}} = p(\text{black}|\text{s}) \\
\text{TPR} &= \text{true positive rate} = \frac{\text{TP}}{\text{TP+FN}} = p(\text{white}|\text{s}) \qquad \text{(S.18)}
\end{aligned}$$

The accuracy is defined as

$$\text{ACC} = \frac{\text{TP + TN}}{\text{TP+TN+FP+FN}} \tag{S.19}$$

and can be estimated through:

$$1 - ACC \approx p(\text{white}|\text{bg}) fBg + p(\text{black}|\text{s})(1 - fBg) \tag{S.20}$$

where fBg is the ratio of background pixels, which we estimate through $fBg = (n_{bg} + 1)/(m + 2)$. Moreover, we have that the false discovery rate (fraction of of false positives among positives) is

$$\text{FDR} = \frac{\text{FP}}{\text{FP + TP}} \approx \frac{n_{bg} \cdot p(\text{white}|\text{bg})}{n_{white}} \tag{S.21}$$

The false omission rate (fraction of false negatives among negatives) is

$$\text{FOR} = \frac{\text{FN}}{\text{FN + TN}} \approx \frac{n_{\text{black}} - n_{bg} \cdot p(\text{black}|\text{bg})}{n_{black}}. \tag{S.22}$$

Two more common measures are the false discovery rate

$$\text{FDR} = \frac{\text{FP}}{\text{FP +TP}} \tag{S.23}$$

and the false omission rate

$$\text{FOR} = \frac{\text{FN}}{\text{FN +TN}}. \tag{S.24}$$

S7: Estimating λ_{bg} and N_{icr}^{bg} using truncated fits and a goodness-of-fit test procedure

In the Methods section in the main text, we introduce our procedure for estimating the Poisson parameter, λ_{bg} for background regions in an image and the "optimal" truncation point, N_{icr}^{bg}. Here, we illustrate the output of our procedure for synthetic images and for the two experimental images in the main text.

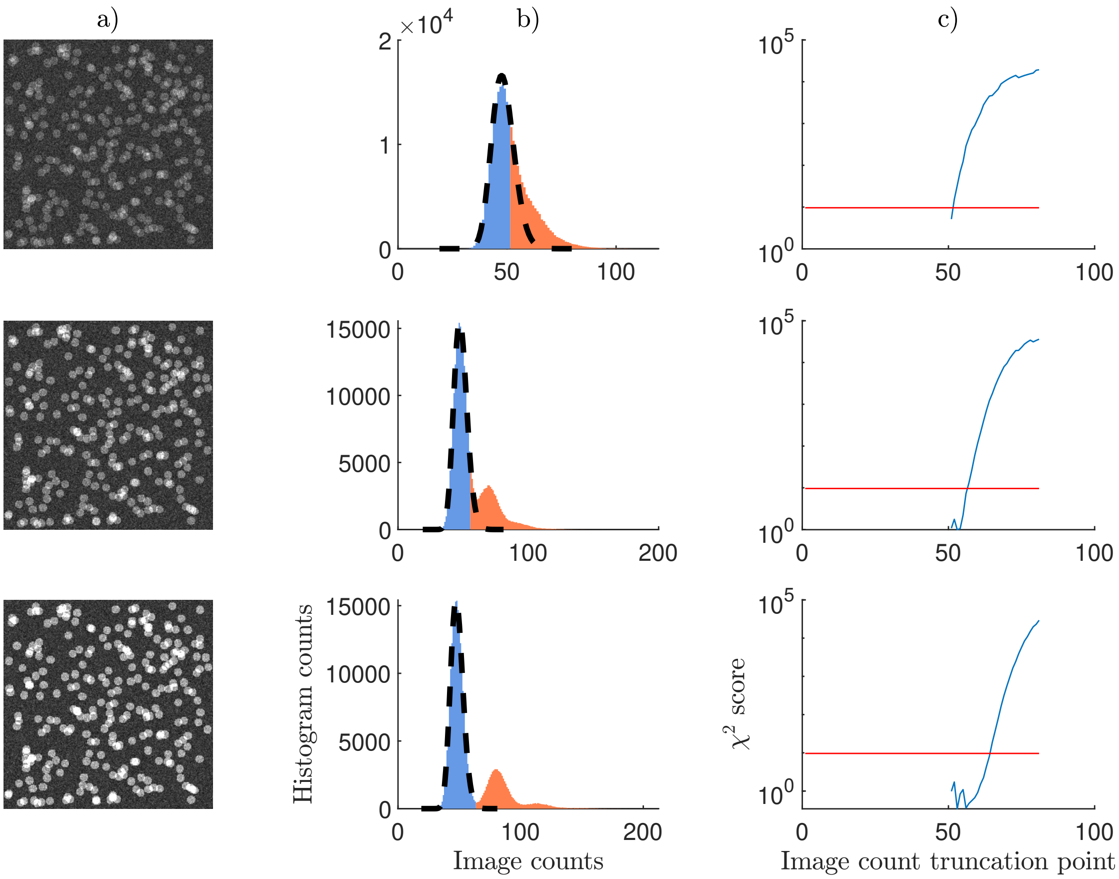

Fig S4. Estimation of λ_{bg} and N_{icr}^{bg} for synthetic images of varying SNR ratios. a) Synthetic images (see Sec. S4). b) Image count histograms for the images in panel a) with an overlaid fitted EMCCD-PMF with λ_{bg} and truncation points as fit parameters. c) χ^2 scores for different image count truncation points. Here, we used 5 bins when calculating the χ^2 score, with bin edges determined by largest image count values for which the histogram of all image counts up to the edge were lower than 0.2, 0.4, 0.6 and 0.8 quantile. When the χ^2 values are below the horizontal lines, the fit is deemed as good (here, with a goodness-of-fit p-value, $p_{GoF} = 0.01$). As "optimal" truncation point, N_{icr}^{bg}, we use the largest truncation point which passed the goodness-of-fit test. We used the following SNR values: top row, SNR = 3, middle row, SNR = 4.5 and bottom row, SNR = 6 when generating the synthetic images.

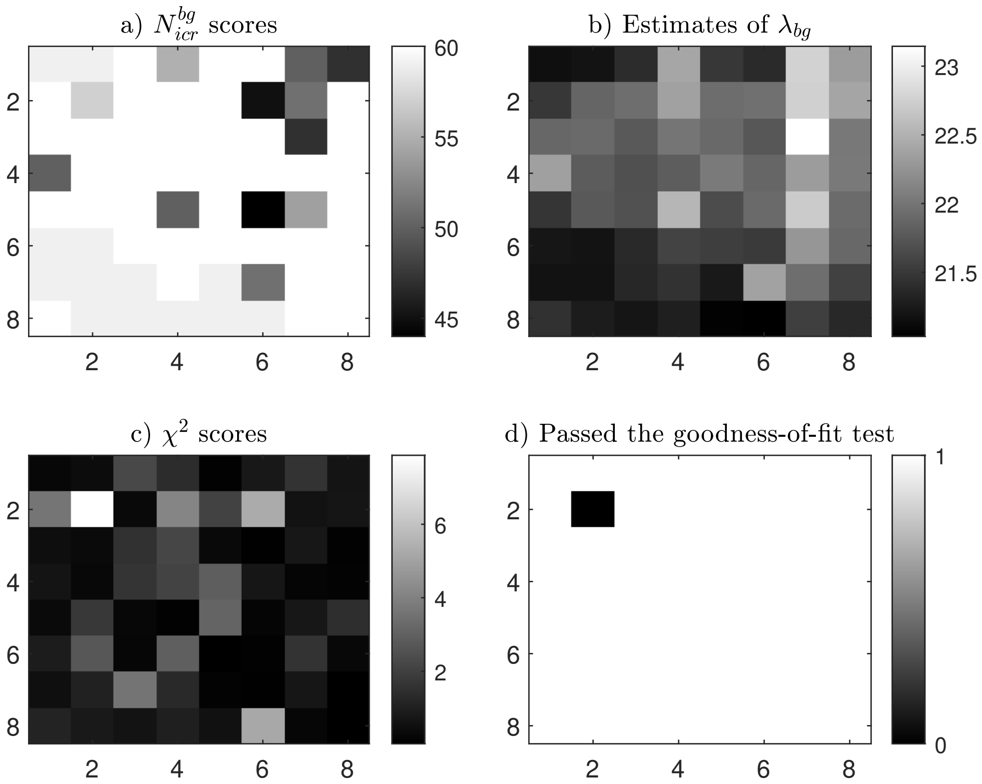

Fig S5. Estimates of N_{icr}^{bg} and λ_{bg} as well as goodness-of-fit statistics for the for the experimental image of fluorescent beads (Figure 1 in the main text). (a) Estimated value for the "optimal" threshold, N_{icr}^{bg} for each of the tiles in Figure 1 in the main text. (b) Estimates of λ_{bg} with a truncation point at N_{icr}^{bg}. (c) Goodness-of-fit χ^2 scores with a truncation point at N_{icr}^{bg}. d) Tiles which passed the χ^2 test at 1% significance level. Here, a value = 1 correponds to a passed test, and a value = 0 indicate that the test was not passed.

S8: Image count histogram for the experimental lung cancer data

In Figs. S7 and S8 we provide additional results associated with Figure 3 in the main text.

S9: Miscellaneous supplementary plots

Figs. S9 and S10 provide further examples of our binarization and segmentation methods.

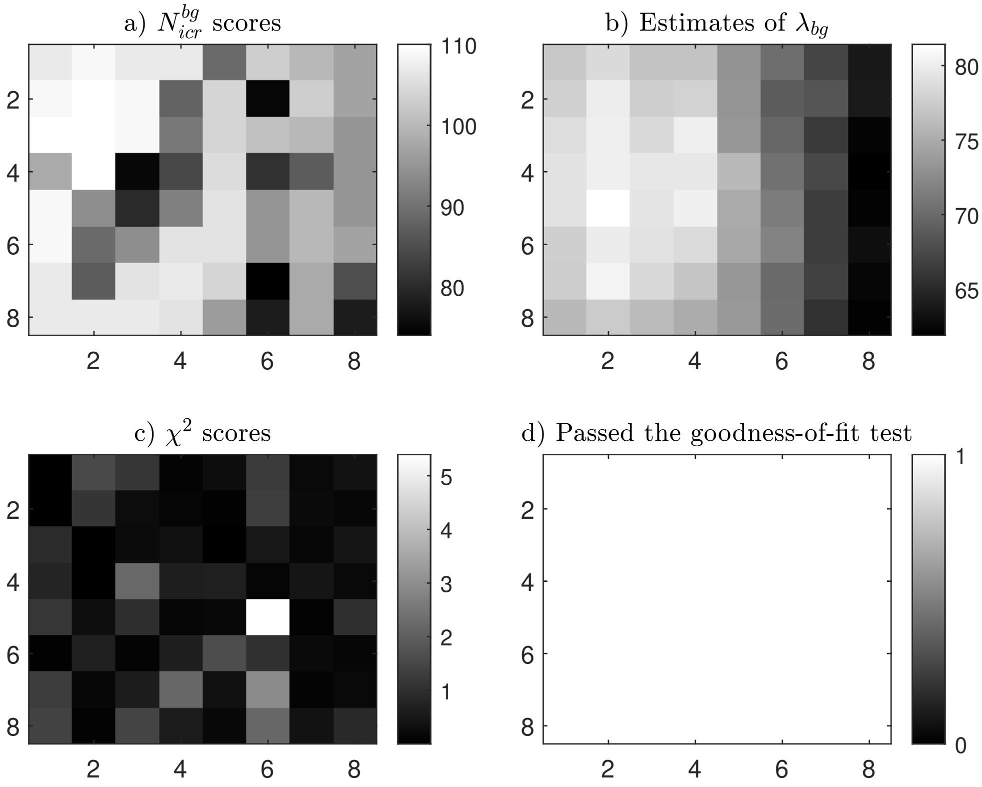

Fig S6. Estimates of N_{icr}^{bg} and λ_{bg} as well as goodness-of-fit statistics for the experimental lung cancer image (Figure 3 in the main text). This figure has identical panels as in Fig. S5: (a) Estimated value for N_{icr}^{bg} for each of the tiles in Figure 3 in the main text. (b) Estimates of λ_{bg}. (c) Goodness-of-fit χ^2 scores at the optimal image count threshold. d) Tiles which passed the χ^2 test at 1% significance level.

References

1. Abariute L, Lard M, Hebisch E, Prinz CN. Uptake of nanowires by human lung adenocarcinoma cells. Plos one. 2019 Jun 21;14(6):e0218122.

2. Sage D, Pham TA, Babcock H, Lukes T, Pengo T, Chao J, Velmurugan R, Herbert A, Agrawal A, Colabrese S, Wheeler A. Super-resolution fight club: assessment of 2D and 3D single-molecule localization microscopy software. Nature methods. 2019 May;16(5):387-95.

3. Witkovsky V. Numerical inversion of a characteristic function: An alternative tool to form the probability distribution of output quantity in linear measurement models. Acta IMEKO. 2016 Nov 4;5(3):32-44.

4. Reibel, Y., Jung, M., Bouhifd, M., Cunin, B., Draman, C. (2003). CCD or CMOS camera noise characterisation. The European Physical Journal-Applied Physics, 21(1), 75-80.

5. Gil-Pelaez J. Note on the inversion theorem. Biometrika. 1951 Dec 1;38(3-4):481-2.

6. Davies, R. B. (1973). Numerical inversion of a characteristic function. Biometrika, 60(2), 415-417.

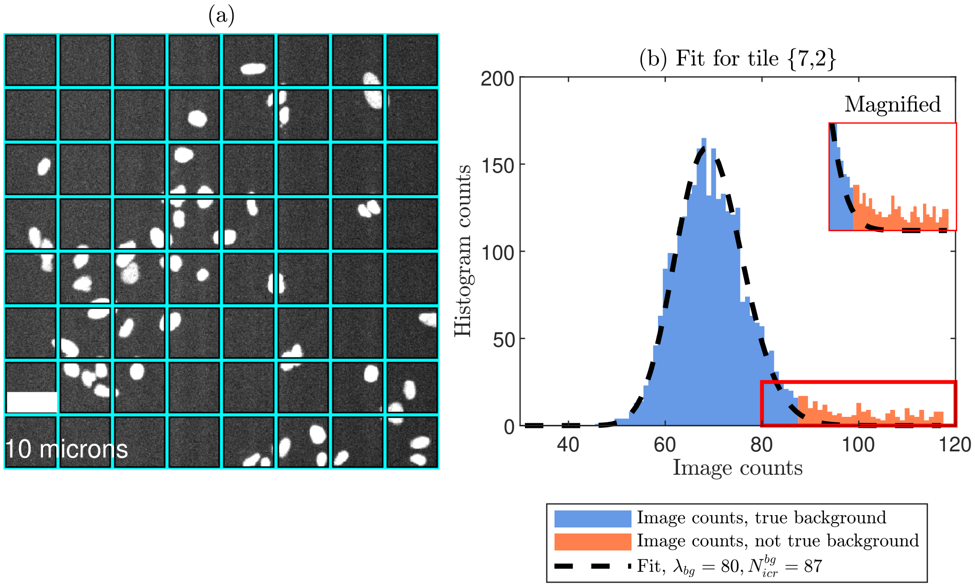

Fig S7. Background Poisson parameter estimation and image thresholding for the lung cancer cell images. (a) Lung cancer cell image example. (b) Image count histogram with an overlaid fit obtained using p-value threshold, $p_{GoF} = 0.01$.

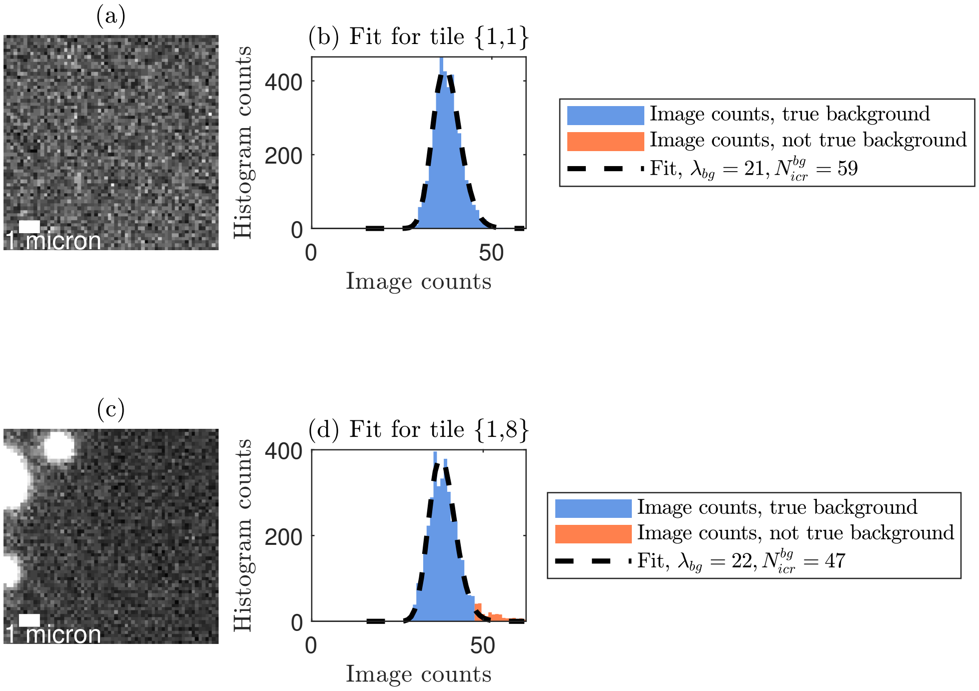

Fig S8. Estimating λ_{bg} for an image which contains both background and signal. (a,c) Tiles of the experimental fluorescence microscopy image of fluorescent beads. The contrast is set to better display the background noise and glass slide artefacts. (b,d) Estimating λ_{bg}: A histogram of the image counts for a single tile. The blue bars represent pixels regarded as true background, while the orange bars represent the outliers (not true background or signal pixels). The image counts threshold, separating the blue and orange bars was determined using a p-value threshold, $p_{GoF} = 0.01$, for the goodness-of-fit tests. The dashed black curve shows the fitted PMF for the estimated λ_{bg}, extended to the full range of image counts (in our method, we fit a truncated PMF to the blue bars)

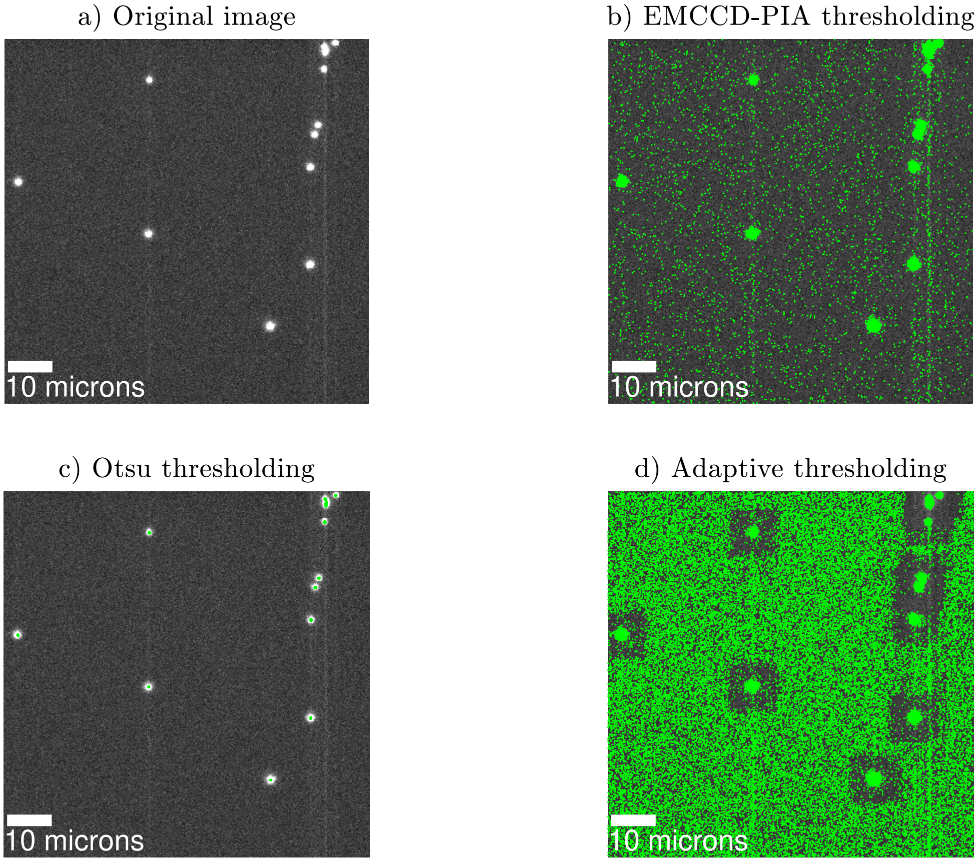

Fig S9. Comparison of thresholding methods for image binarization. (a) Original fluorescent beads image from the main text (Fig 3, panel a). (b) Fluorescent beads image overlayed with binarized image using EMCCD-PIA. The green pixels represent the pixels classified as white pixels. We set the p-value binarization threshold as $p_{binarize} = 0.01$, which yields roughly 1 % false positives as it should. (c) Fluorescent beads image with detected regions using Otsu thresholding. (d) Fluorescent beads image with detected regions using Matlab's adaptive thresholding function (`adaptthresh`) with default sensitivity (0.5). Notice that Otsu underestimates the size of the beads, and adaptive thresholding needs sensitivity parameter to be tuned in order to get accurate thresholding. In contrast, in our method we have excellent control over false positives. The binarized image in panel b) is used as input to our image segmentation method (see main text).

a) b)

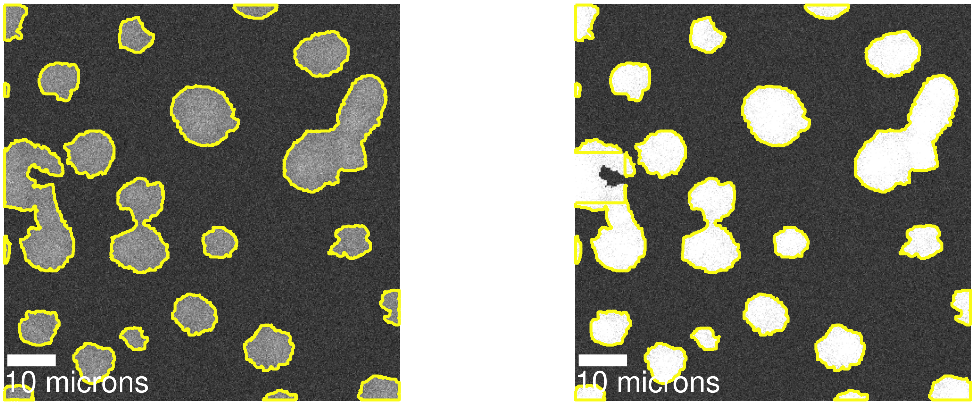

Fig S10. Photophysical image segmentation for a synthetic image with objects of complicated geometries. (a) Synthetic cell image (SNR = 3.5) with detected regions (connected components of white pixels). The original image can be found in https://blogs.mathworks.com/images/steve/60/nuclei.png (courtesy of Dr. Ramiro Massol). The yellow pixels are boundary points to the detection regions. (b) Synthetic cell image (SNR = 7.5) with detected region. We here tile the image and set p-value thresholds $p_{GoF} = 0.01$ and $p_{\text{binarize}} = 0.01$ and region size gap threshold = 1. Notice that our image segmentation method performs visually well on both experiments. To get a synthetic cell image for any choice of the SNR, the original image was first binarized using Contrast-limited Adaptive Histogram Equalization (CLAHE), Otsu thresholding, and morphological operations to remove small regions. The resulting black/white image was processed as in Section S4.
